# Emerging role of quantitative imaging (radiomics) and artificial intelligence in precision oncology

**DOI:** 10.37349/etat.2023.00153

**Published:** 2023-08-24

**Authors:** Ashish Kumar Jha, Sneha Mithun, Umeshkumar B. Sherkhane, Pooj Dwivedi, Senders Puts, Biche Osong, Alberto Traverso, Nilendu Purandare, Leonard Wee, Venkatesh Rangarajan, Andre Dekker

**Affiliations:** University of Campania “L. Vanvitelli”, Italy; ^1^Department of Radiation Oncology (Maastro), GROW School for Oncology, Maastricht University Medical Centre+, 6200 Maastricht, The Netherlands; ^2^Department of Nuclear Medicine, Tata Memorial Hospital, Mumbai 400012, Maharashtra, India; ^3^Homi Bhabha National Institute, BARC Training School Complex, Anushaktinagar, Mumbai 400094, Maharashtra, India; ^4^Department of Nuclear Medicine, Advance Center for Treatment, Research, Education in Cancer, Kharghar, Navi-Mumbai 410210, Maharashtra, India

**Keywords:** Precision oncology, radiomics, imaging biomarkers, artificial intelligence

## Abstract

Cancer is a fatal disease and the second most cause of death worldwide. Treatment of cancer is a complex process and requires a multi-modality-based approach. Cancer detection and treatment starts with screening/diagnosis and continues till the patient is alive. Screening/diagnosis of the disease is the beginning of cancer management and continued with the staging of the disease, planning and delivery of treatment, treatment monitoring, and ongoing monitoring and follow-up. Imaging plays an important role in all stages of cancer management. Conventional oncology practice considers that all patients are similar in a disease type, whereas biomarkers subgroup the patients in a disease type which leads to the development of precision oncology. The utilization of the radiomic process has facilitated the advancement of diverse imaging biomarkers that find application in precision oncology. The role of imaging biomarkers and artificial intelligence (AI) in oncology has been investigated by many researchers in the past. The existing literature is suggestive of the increasing role of imaging biomarkers and AI in oncology. However, the stability of radiomic features has also been questioned. The radiomic community has recognized that the instability of radiomic features poses a danger to the global generalization of radiomic-based prediction models. In order to establish radiomic-based imaging biomarkers in oncology, the robustness of radiomic features needs to be established on a priority basis. This is because radiomic models developed in one institution frequently perform poorly in other institutions, most likely due to radiomic feature instability. To generalize radiomic-based prediction models in oncology, a number of initiatives, including Quantitative Imaging Network (QIN), Quantitative Imaging Biomarkers Alliance (QIBA), and Image Biomarker Standardisation Initiative (IBSI), have been launched to stabilize the radiomic features.

## Introduction

Cancer is a disease that develops when body cells don’t follow the cell signalling and proliferate uncontrollably [1–4]. Cancerous cells have the ability to spread to distant regions of the body, invade nearby tissues, or even do both. Cancer is considered a genetic disease due to the alterations that occur in the genes responsible for regulating various aspects of cell behavior, particularly their division, and development. These genetic changes disrupt the normal functioning of cells, leading to uncontrolled growth and the formation of tumors. The body usually eliminates these genetically altered cells before they can turn cancerous [1–4]. But on several occasions, the body is unable to do so, most frequently as people age, the body grows less capable of destroying these altered cells. This increases the likelihood of acquiring cancer in later life. Every cancer is unique due to the genetic alterations present in it. There are more than 100 types of cancer present which may be categorized as carcinomas, sarcomas, lymphomas, myelomas, multiple myeloma, leukaemia, brain and spinal cell tumours, germ cell tumours, neuroendocrine tumours, and carcinoid tumours [1–4].

According to Globocan 2018, 18.1 million new instances of cancer are reported each year worldwide [5]. In 2020, it was predicted that there would be 10.3 million cancer deaths and 19.3 million new cases of cancer worldwide. It is frightening that there have been 1.2 million more cancer diagnoses globally in the last two years [6]. Nearly 9.5 million (49.2%) of the 19.3 million total new cases come from Asia alone followed by Europe (22%), America (13.3%), Latin America and the Caribbean (7.6%), and Africa (5%) [6]. Breast cancer (11.7%), lung cancer (11.1%), and colorectal cancer (10.1%) are the three most common cancers worldwide. According to the Globocan 2020 report, breast cancer is the most prevalent cancer in women (24.5%) and lung (14.3%) and prostate (14.1%) cancer are the most prevalent in men worldwide [6]. This article aims to review the role of radiomics and artificial intelligence (AI) in precision oncology.

## Cancer treatment and the role of imaging

**Figure 1:**
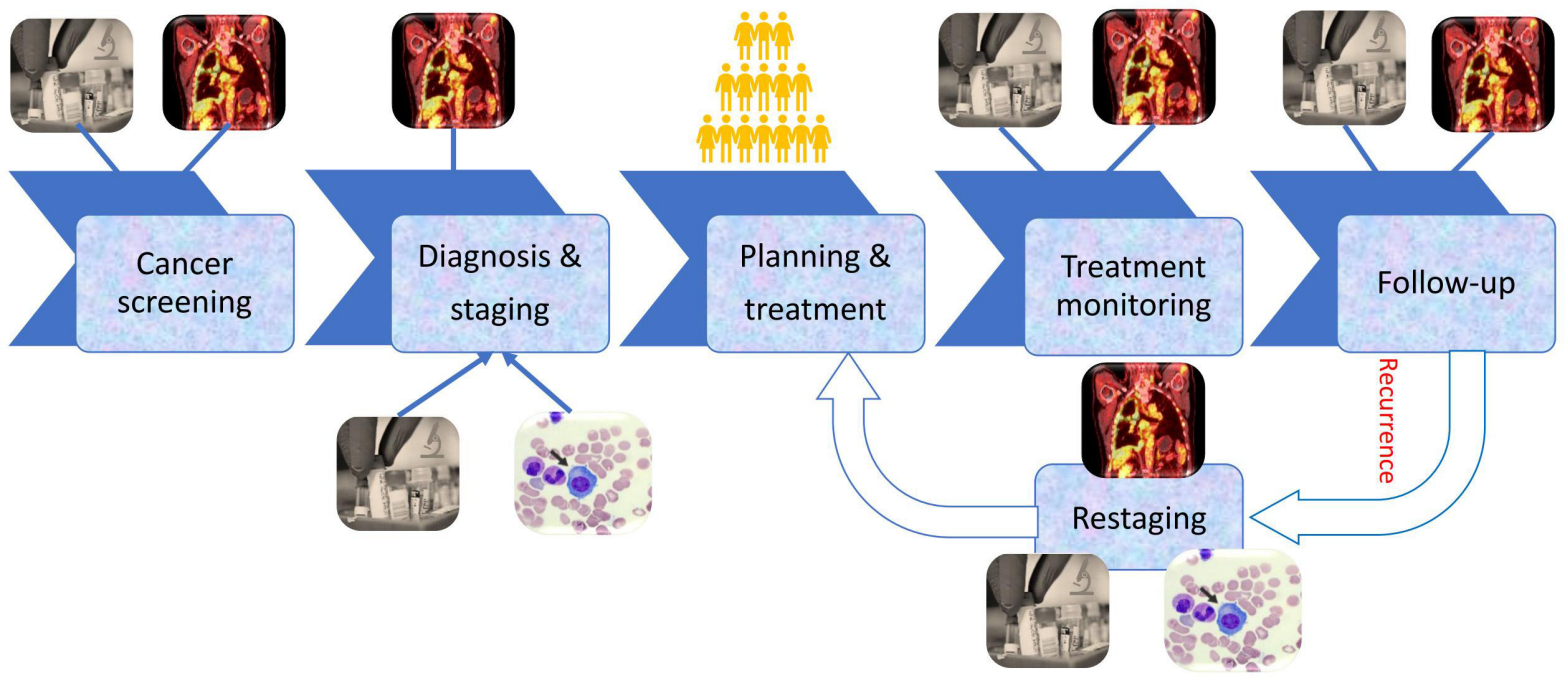
Various stages of the cancer detection and treatment process

Treatment of cancer is not straightforward; it is a complex and long process [7–9]. The cancer treatment process starts with the diagnosis of the disease and in some cases never ends while the patient is alive. The process of cancer detection and treatment and the role of imaging is summarized in **Figure 1**. Screening/diagnosis of the disease is the beginning of cancer management and continued with the staging of the disease, planning and delivery of treatment, treatment monitoring, and ongoing monitoring and follow-up.

Although pathology is the confirmatory diagnosis of cancer, imaging plays a significant role in the diagnosis of cancer [7–9]. Simple imaging modalities like an X-ray, optical imaging, mass spectrometry, and photoacoustic imaging to complex modalities like sonography, computed tomography (CT), single-photon emission computed tomography (SPECT), positron emission tomography (PET), and magnetic resonance imaging (MRI) are used for the diagnosis of cancer [7–15]. These modalities are used for the localization and characterization of the disease. For instance, a chest X-ray is often performed for the initial identification and localization of lung cancer. A mammography X-ray is performed for diagnosis of breast cancer. Ultrasound is widely used for (additional) diagnosis of breast cancer, prostate cancer, and gynaecological cancers [7–12]. MRI and CT are helpful in the diagnosis of head and neck cancer and bone, soft tissue, and brain tumours [12–15]. PET/CT is widely used for the diagnosis of lymphomas and many other malignant conditions to detection of distant metastases [16–18]. Often in parallel with diagnosis, imaging is being used to stage disease [19]. This complex process includes consideration of the primary tumour, nodal metastasis (TNM), and distance metastasis. Based on imaging and pathology findings, the TNM staging is performed which results in a final overall stage of the disease which to a larger extent determines the treatment [7–9], and it typically requires a multidisciplinary approach with surgery, radiotherapy or chemotherapy, or a combination of these often considered as the best treatment [5–9]. In the diagnosis, staging, and treatment of cancers, the role of imaging is extensive [10–24]. For radiotherapy planning, contrast-enhanced CT is performed which is called planning CT. Planning CT is utilized to plan the treatment using external beam radiotherapy (EBRT) or brachytherapy (BT). In a radiotherapy treatment planning system (TPS), treatment is simulated by using the planning CT for advanced radiotherapy equipment like tomotherapy, image-guided radiotherapy (IGRT), and intensity-modulated radiotherapy (IMRT) [24]. Planning CT scans are ready-to-use medical images for radiomic extraction as it has the tumour delineation by an expert in the form of gross tumour volume (GTV) [25, 26].

Treatment monitoring and follow-up are performed to assess the efficacy of the treatment and related side effects of the treatment. Monitoring of patients is performed by checking the vitals of the patients, blood parameters, and changes in imaging parameters. Most imaging parameters are used to monitor the regression or progression of disease during and after treatment [5–7, 15–17, 22–24].

Post-treatment follow-up is performed to identify the general status of the patient post-treatment, the condition of the disease, and the recurrence or progression of the disease [16–21]. Various blood parameters are used to check the general condition and progression of the disease. Imaging parameters are used to identify the disease condition, i.e., stable disease, local recurrence, and nodal and distant metastasis [7–9].

Many times, during the follow-up we encounter the recurrence of the disease. Treatment may be warranted in case of recurrence. In this scenario, the restaging of the disease is performed based on imaging and pathological finding, and subsequently, suitable treatment is offered [5–9, 21].

Imaging plays a significant role in the management of cancer. Imaging is one of the most important diagnostic modalities which indicates the onset of cancer and recurrence during the follow-up.

Various types of imaging can be used for the management of cancer, i.e., X-ray, ultrasound, CT, MRI, SPECT, and PET [20–24]. CT, MRI, PET, and SPECT are performed during staging, restaging, and follow-ups [10–13, 20–24]. Multi-modality imaging like PET/CT, SPECT/CT, and PET/MRI is being considered more and more in cancer management. This manuscript reviews the role of quantitative imaging and AI in oncology.

## Quantitative imaging

Image findings can be categorized into qualitative and quantitative parameters that are helpful in diagnosis and cancer management. Qualitative parameters are subject to interpreter variability whereas quantitative parameters are independent of interpreter variability [27]. In current care, imaging data is often analyzed qualitatively and semi-quantitatively to diagnose and stage the pathological condition. With visual perception and semi-quantitative data, only limited information is extracted from the image.

But information stored in medical images may not be amenable to visual interpretation by expert human eyes. This information can be extracted using mathematical and statistical formulas as various quantitative parameters which may be helpful in disease stratification and prognostication in cancer [25]. Quantitative imaging is the conversion of images into quantitative features that may be associated with important outcomes such as tumour response to treatment in cancer management [26].

Conventional quantitative parameters like the difference in Hounsfield unit (HU) tumour size measurement in CT, the difference in standardized uptake value (SUV) value or uptake value in PET, the difference in proton density, and diffusion coefficient and spectral peak in MRI have demonstrated the capabilities to differentiate between the responders and non-responders in cancer therapies [26–28].

For example, response evaluation criteria in solid tumours (RECIST) [29, 30] is used to assess the treatment response on CT and similarly, PET response criteria in solid tumours (PERCIST) [31, 32] is used to assess the treatment response on PET. RECIST assesses the response of treatment based on size regression or tumour which amounts to an anatomical response whereas PERCIST is based on the change in SUV which is considered a physiological response [31–35].

## Radiomics

Radiomics moves beyond these conventional parameters and applies quantitative imaging in a much broader context, e.g., by extracting many more features from the image. This has shown the potential to access the treatment response and outcomes in initial studies [36]. Radiomics, in other words, is a process to extract high throughput quantitative parameters from medical images to unearth various pathological conditions [36–40] that are associated with outcomes.

**Figure 2:**
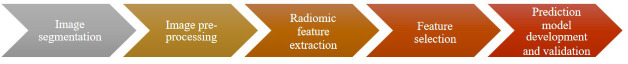
Radiomic workflow

Radiomic features extracted from medical images can be classified as shape-based features, first-order features, higher-order features, textural features, Laplacian-of-Gaussian (LoG) features, and wavelet features [37–41]. The entire radiomic process involves image extraction, tumour segmentation, and pre-processing of images, i.e., conversion of images in the required format, voxel normalization, image masking, filtering of image, image transformation, and radiomic extraction involves extraction of radiomic features from original, filtered, and transformed images (**Figure 2**) [40, 41]. This process leads to data explosion which can be managed by employing feature selection or reduction methods. Finally, extracted/selected features are used for radiomic prediction model development.

## AI and big data

**Figure 3:**
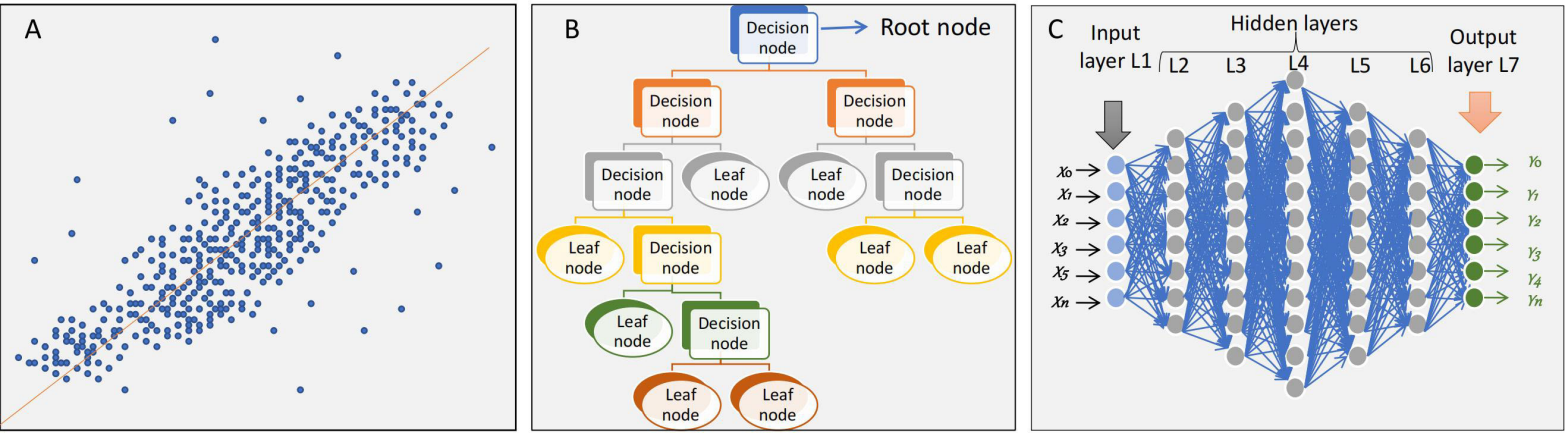
ML algorithms

AI can be defined as the process to develop intelligent machines which can replace or outperform the natural intelligence of human beings [42]. Machine learning (ML) and big data are the two main components of AI. Big data can be defined by 5Vs—volume: the data in large volume; variety: data from various sources; velocity: data grows very fast; veracity: quality and integrity of data; value: the richness of data [43]. ML is the method by which a machine learns from past events or data without being explicitly programmed for that. There are three main types of ML algorithms known (A) regression algorithm, (B) decision tree (DT) algorithm, and (C) deep learning (DL) algorithm (**Figure 3**). These ML algorithms can be trained by using (A) supervised learning, (B) unsupervised learning, and (C) semi-supervised methods [44]. DL is a subtype of ML in which the algorithm learns a composition of features that are represented in a hierarchy of structures in the data. Complex representations of data are expressed in terms of simpler representations of data in DL [45]. Various DL algorithms propose an end-to-end approach to predict outcomes by learning simple features in a hierarchical manner as components of complex features.

There are various kinds of AI algorithms, e.g., linear regression, logistic regression, k-nearest neighbour (KNN), random forest (RF), support vector machine (SVM), Bayesian network (BN), and DL algorithms such as convolutional neural networks (CNNs), recurrent neural networks (RNNs), and artificial neural networks (ANNs) [44–50]. Typically selecting the “right” algorithm depends on the task at hand [51–53]. Hence, prediction algorithms need to be selected wisely and several algorithms need to be tested to select the best algorithm for the prediction problem at hand. Typically, binary or multi-class classification problems are solved using machine learning algorithms like logistic regression, random forest classifiers, support vector classifiers, etc., whereas continuous or discrete outcomes are predicted using algorithms like linear regression, random forest regressors, support vector machine regressors, etc. These algorithms are used to create a number of prediction models depending on the prediction endpoints. The best model is chosen after these models have been evaluated based on the prediction outcome matrices, including accuracy, precision, recall, F1-score, and area under the curve (AUC), using the training and validation datasets.

## Precision oncology

**Figure 4:**
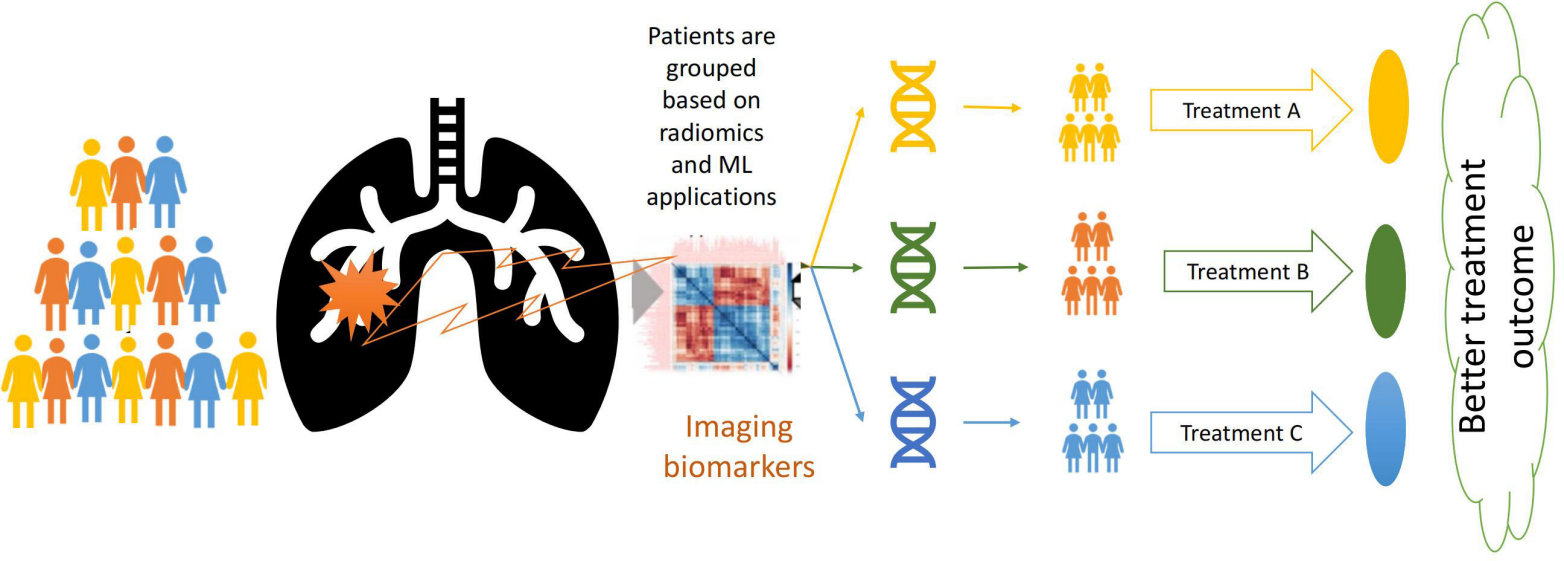
Precision oncology workflow leveraging imaging biomarker

Cancer treatment has advanced significantly over the last several decades in terms of technical developments in diagnostic and therapeutic equipment and also because of the development of various new drugs and newer and more precise surgical techniques. Often clinicians decide on the treatment meticulously considering all factors, i.e., histology of the tumour, stage of the disease, and general condition of the patients [54, 55]. Despite tremendous development in cancer treatment and expertise, treatment success has many variabilities; on several occasions, these treatments fail miserably [56]. This led to the evolution of personalized medicine in oncology [57]. Personalized oncology works on the principle of identification of subgroups of patients in particular disease types and uses specific treatments for the subgroups [57, 58]. Many biomarkers and gene mutations have been investigated to identify the subgroups of patients in various cancers and targeted drugs for those subgroups [58, 59]. In recent years several imaging biomarkers are also being investigated for the identification of such subgroups and to predict the outcome of the treatment (**Figure 4**).

## Limitations of radiomics

An inherent problem of repeatability and reproducibility exists with radiomics which is caused due to the difference in the scanner from different vendors, different acquisition protocols, and intra-scanner variations. In our earlier repeatability and reproducibility study, we found that only 10% of CT radiomic features had good repeatability and reproducibility in clinical cohorts and phantom [60]. Traverso et al. [61] in a systematic literature review have also concluded that there are stability issues with the majority of radiomic features. To harmonize radiomic extraction tools, features, and imaging standards, several initiatives are started by various agencies, like the Quantitative Imaging Network (QIN) [62], the Quantitative Imaging Biomarkers Alliance (QIBA) [63], and Quantitative Imaging in Cancer: Connecting Cellular Processes with Therapy (QuIC-ConCePT) [64]. These agencies are working continuously to standardize imaging and imaging biomarkers. The Image Biomarker Standardisation Initiative (IBSI) is another consortium that works towards the harmonization of radiomic features across the globe by minimizing the deviation in imaging and standardizing the radiomic extraction process [65, 66]. QIN is an initiative to harmonize the imaging parameters and hardware across the vendor. QIBA has initiated scanning phantoms to trace the standard differences between the equipment. The main objective of such a phantom study is to find the errors related to data collection and establish the procedures to harmonize the performance of imaging equipment among different makes and models with the goal to reduce the bias and variance across the equipment. QIN initiative can be an important step towards stratifying patients through accurate measurements of imaging biomarkers [62]. The radiomics quality score (RQS) is another initiative proposed by Lambin et al. [37] to address the issues related to radiomic study reporting. Most of these initiatives will assist in advancing the standardization process of imaging biomarkers.

## AI infrastructure development using the findable, accessible, interoperable, and reusable data concept

A prerequisite for the implementation of AI in hospitals is the transformation of the different data elements [digital imaging and communication in medicine (DICOM), radiomic features, and clinical data, diagnostic, pathology, genetic] into a findable, accessible, interoperable, and reusable (FAIR) format [67]. Data should be organized and archived as such; it should be FAIR for both humans and machines. Hence, the data should be anonymized and assigned global and persistent identifiers. Furthermore, the accessibility aspect includes a standardized protocol (authorization and identification) so that authorized users can easily access the data. The connection of the different data concepts with domain ontologies enables the interoperability between the different researchers interested in using the data, while the reusability is succeeded with the inclusion of rich documentation to support data interpretation for the different users. These data principles are the guidelines to improve the quality of data holdings and focus on enhancing the automation to find and reuse the data.

Oncology treatment produces a huge amount of data that satisfies all 5Vs of big data [68]. Those data are either stored in various files in the medical record section of the hospital or in the form of free text or tables in the hospital information system (HIS) which are not amenable to ML. Harvesting and transforming those data in the machine-understandable form is challenging for a data scientist [26]. There is a need to develop an automated system to transform and store these medical records in a format amenable to ML.

## Radiomics and precision oncology

Various studies have been performed to demonstrate the utility of radiomics in cancer management [59, 68]. Radiomics-based studies have witnessed rapid growth in the last decade; several studies have been published showing the potential of radiomics in diagnosing and treating cancer. Many radiomic-based AI decision support systems (DSSs) have been developed in oncology and reported in the literature. In the last few years, new aspects of radiomics such as delta radiomics are being researched [69]. Delta radiomics comprises the extraction and comparison of quantitative features from sequential scans acquired throughout treatment, which provides information on the efficacy of the treatment [69, 70]. The utility of radiomic-based prediction modelling has been tested widely in diagnosing and treating all varieties of solid tumours. Literature is suggestive of the utilization of radiomic features as quantitative biomarkers for several oncological conditions like brain tumours, head-and-neck cancer, breast cancer, lung cancer, colorectal cancer, prostate cancer, gastrointestinal (GI), liver cancer, and cervical cancer [70–94].

Wang et al. [77] in their study have developed a response prediction model for induction chemotherapy using MRI radiomic features. In another study, Sanduleanu et al. [78] developed a tumour hypoxia prediction model combining CT and PET radiomic features. In a similar kind of study, Tran et al. [79] developed a neoadjuvant chemotherapy response prediction model for breast cancer using MRI radiomic features. Park et al. [80] developed a disease-free survival prediction model for breast cancer using MRI radiomic features. Zhang et al. [81] developed a radiomic signature to predict epidermal growth factor receptor (EGFR) mutation in non-small cell lung cancer. Huang et al. [84] developed a disease-free survival prediction model in early-stage (I or II) non-small cell lung cancer using CT radiomic features. Liu et al. [85] in their study, developed a radiomic-based prediction model to predict the response of neoadjuvant chemoradiotherapy in locally advanced rectal cancer. A study by Shiradkar et al. [88] developed MRI based radiomic model to predict biochemical recurrence in the prostate. Altazi et al. [92] have developed a CT radiomic prediction model to predict treatment outcomes in cervical cancer. Reuzé et al. [93] predicted the recurrence of cervical cancer using a PET radiomic-based prediction model. In a study, Chiappa et al. [94] utilized transvaginal ultrasonography radiomics and serum cancer antigen 125 (CA-125) level to develop a DSS to predict the risk of malignancy of ovarian masses. As radiomics features have been identified as potential digital markers in precision oncology, the next goal should be to harmonize the parameters and identify robust radiomic features as potential digital biomarkers.

## Future roadmap

**Figure 5:**
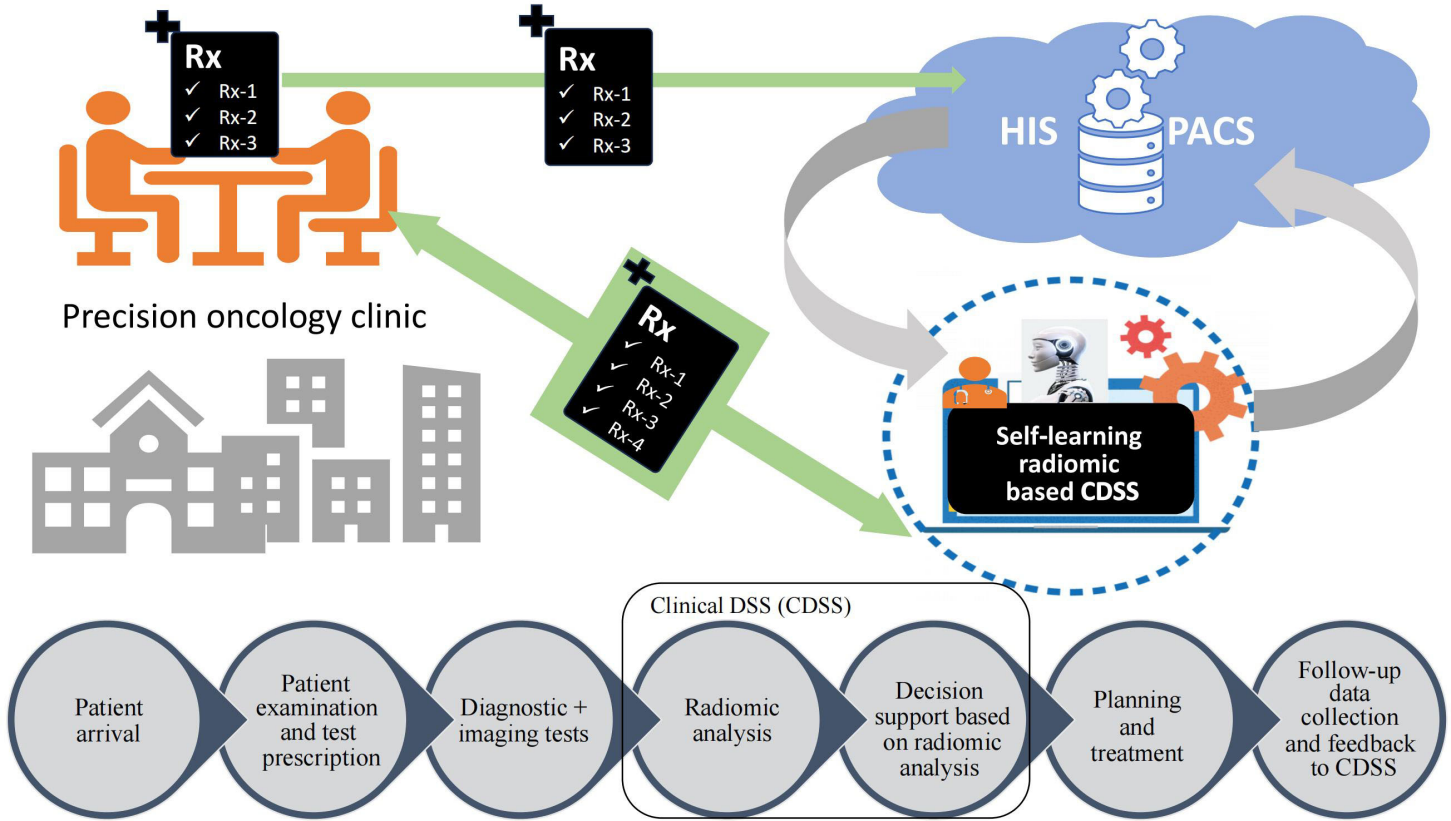
Implementation of self-learning radiomics based clinical DSS in prediction oncology practice. Rx: medical prescription

The future of radiomics lies in the clinical application and implementation of radiomics. A self-learning model may be developed and implemented in the clinic for participation in the DSS. There will be requirements for a super-specialized model to address the specific clinical questions. As suggested by Lambin et al. [37], the image archival system, i.e., picture archiving and communication system (PACS) has to be modified to picture archiving and radiomics knowledge system (PARKS) to store radiomic signatures. The future implementation of the AI-based DSS will help clinicians to improve their decision-making (**Figure 5**).

## Conclusions

The quantitative analysis of medical images (radiomics) has led to the data explosion which is the source of big data in oncology. AI algorithms like ML and DL have been applied to imaging big data to develop DSSs in precision oncology. Several radiomic features have been identified as digital phenotypes of the disease. Nevertheless, several radiomic features have shown the potential to predict various clinical endpoints in oncology, but the translation of these radionics-based prediction models as DSSs in the clinic will require addressing several key issues. The radiomic community needs to address key issues like (A) the robustness of radiomic features, (B) the implementation of AI infrastructure in hospitals, and (C) multi-centre and prospective radiomics studies. The current literature review is suggestive of the role of radiomics and artificial intelligence in precision oncology. We envision that radiomics and artificial intelligence are going to play a pivotal role in phenotyping cancer and guiding cancer management to provide more precise treatments to patients in a true clinical environment soon.

## Abbreviations

CT: computed tomography
DL: deep learning
DSSs: decision support systems
ML: machine learning
MRI: magnetic resonance imaging
PET: positron emission tomography
QIN: Quantitative Imaging Network
SPECT: single-photon emission tomography

## Declarations

### Copyright

© The Author(s) 2023.
